# Enhancement of the Desorption Properties of LiAlH_4_ by the Addition of LaCoO_3_

**DOI:** 10.3390/ma16114056

**Published:** 2023-05-29

**Authors:** Noratiqah Sazelee, Nurul Amirah Ali, Mohammad Ismail, Sami-Ullah Rather, Hisham S. Bamufleh, Hesham Alhumade, Aqeel Ahmad Taimoor, Usman Saeed

**Affiliations:** 1 Energy Storage Research Group, Faculty of Ocean Engineering Technology and Informatics, Universiti Malaysia Terengganu, Kuala Nerus 21030, Terengganu, Malaysia; atiqahsazelee19@gmail.com (N.S.); nurulllamirah@gmail.com (N.A.A.); 2 Department of Chemical and Materials Engineering, King Abdulaziz University, P.O. Box 80204, Jeddah 21589, Saudi Arabia; rathersami@kau.edu.sa (S.-U.R.); hbamufleh@kau.edu.sa (H.S.B.); halhumade@kauedu.sa (H.A.); ataimoor@kau.edu.sa (A.A.T.); umsaeed@kau.edu.sa (U.S.)

**Keywords:** LiAlH_4_, LaCoO_3_, solid-state hydrogen storage, metal oxide

## Abstract

The high hydrogen storage capacity (10.5 wt.%) and release of hydrogen at a moderate temperature make LiAlH_4_ an appealing material for hydrogen storage. However, LiAlH_4_ suffers from slow kinetics and irreversibility. Hence, LaCoO_3_ was selected as an additive to defeat the slow kinetics problems of LiAlH_4_. For the irreversibility part, it still required high pressure to absorb hydrogen. Thus, this study focused on the reduction of the onset desorption temperature and the quickening of the desorption kinetics of LiAlH_4_. Here, we report the different weight percentages of LaCoO_3_ mixed with LiAlH_4_ using the ball-milling method. Interestingly, the addition of 10 wt.% of LaCoO_3_ resulted in a decrease in the desorption temperature to 70 °C for the first stage and 156 °C for the second stage. In addition, at 90 °C, LiAlH_4_ + 10 wt.% LaCoO_3_ can desorb 3.37 wt.% of H_2_ in 80 min, which is 10 times faster than the unsubstituted samples. The activation energies values for this composite are greatly reduced to 71 kJ/mol for the first stages and 95 kJ/mol for the second stages compared to milled LiAlH_4_ (107 kJ/mol and 120 kJ/mol for the first two stages, respectively). The enhancement of hydrogen desorption kinetics of LiAlH_4_ is attributed to the in situ formation of AlCo and La or La-containing species in the presence of LaCoO_3_, which resulted in a reduction of the onset desorption temperature and activation energies of LiAlH_4_.

## 1. Introduction

Due to the world’s population growth and civilizational developments, there has been an exponential increase in energy demand. The exploration of sustainable and renewable energy such as wind energy, hydropower, electrochemical energy and solar energy is believed to relieve the burden on the current energy demand [1,2,3]. Moreover, the amount of renewable energy consumed today is increasing which raises the standard for energy storage and transportation. However, the primary energy source is still fossil fuels despite the fact that they are unsustainable and have brought serious problems to the environment and human health [4,5]. Based on these facts, all countries have begun to implement a variety of strategies in order to avoid such problems. Future carbon emissions are predicted to be significantly reduced by hydrogen [6,7]. Over the last two decades, studies on hydrogen storage have received more attention in the literature [8,9]. In addition, hydrogen has the tremendous benefit of providing clean secondary energy with high energy content, no pollution and abundant resources [10,11]. There are three ways of storing hydrogen: (i) in a liquid form [12], (ii) in compressed gas [13] and (iii) in solid-state forms [14]. However, storing hydrogen in a solid-state form is attractive due to its safety reasons. The solid-state form has two main types: (i) physisorption and (ii) chemisorption. In physisorption, the hydrogen bind in the molecular to the surface of the host materials through weak interaction [15]. This physisorption process occurs for materials such as metal-organic frameworks [16] and carbon-based materials [17]. In 2023, a study by Mishra and colleagues [18] explored novel polycrystalline carbon nanotubes (PCNTs) in order to be used for large-scale hydrogen storage applications. They also revealed that PCNTs with moderate grain size are quite effective for these applications. Liang et al. [19] also reported an effective way to enhance hydrogen storage performance by using a porous carbon nanotube (CNT). However, the physisorption process has a low enthalpy of adsorption as revealed by previous studies [20,21]. Chemisorption is favorable because it can create a better storage capacity in ambient conditions [22]. Complex hydrides (such as NaBH_4_ and LiAlH_4_) and metal hydrides (such as MgH_2_) are typical materials for chemisorption. Other typical materials for chemisorption in chemical hydrogen storage are ammonia borane, methane, dimethyl ether, methanol, methanol and formic acid [23]. However, these chemical hydrides materials also suffer from some drawbacks, for example, ammonia borane (NH_3_BH_3_) requires a long induction time and high temperature to release hydrogen [24] and ammonia (NH_3_) must be heated at high temperature (above 650 °C) to achieve complete conversion [25].

In this study, lithium aluminium hydride (LiAlH_4_), which are complex hydrides, have been used due to their low cost, moderate temperature to release hydrogen and high hydrogen storage capacity (10.5 wt.%) [26,27]. However, the use of LiAlH_4_ for hydrogen storage applications is still constrained by its sluggish kinetics and irreversibility [28,29]. Furthermore, LiAlH_4_ needs to be handled according to the safety data sheet and Standard Operating Procedure (SOP). LiAlH_4_ needs to be stored away from heat/flame and moisture/water sources and kept sealed under an inert atmosphere. Personal protective equipment including safety glasses, lab coat, gloves and covered shoes should be used. In order to overcome the disadvantages, a few techniques for enhancing its kinetics and thermodynamics have been investigated. For example, doping LiAlH_4_ with various kinds of catalysts or additives such as metal oxides [30,31,32,33], metal halides [34,35], metal hydrides [36], pure metals [37,38] and carbon material [39] have been performed via the ball-milling method. However, the improvement of LiAlH_4_ is adversely affected by the ball-milling technique as proposed by Resan and colleagues [40].

A study by Jiao and colleagues [41] revealed that excellent desorption properties of LiAlH_4_ were accomplished after being doped with 1 wt.% NiCo nanoalloy encapsulated in graphene layers (NiCo@G). The starting hydrogen release for the doped samples was unexpectedly lowered by 109 °C compared to pure LiAlH_4_ (152 °C). For the desorption kinetic performance, as-milled LiAlH_4_ releases 1.6 wt.% of H_2_ at 150 °C within 10 min. Meanwhile, faster desorption kinetics can be observed after LiAlH_4_ is doped with 1 wt.% NiCo@G. The desorption kinetics for the doped samples carries on rapidly with 5.8 wt.% of H_2_ under the same conditions. As investigated by Li et al. [42], the addition of CoFe_2_O_4_ nanopowder to LiAlH_4_ effectively lowered the decomposition kinetic barrier for LiAlH_4_. Additionally, the activation energies of LiAlH_4_ doped with CoFe_2_O_4_ for the first two stages were 52.4 kJ/mol and 86.5 kJ/mol, respectively. During the heating process of LiAlH_4_ doped with CoFe_2_O_4_, the LiAlO_2_, LiFeO_2_, Al_0.52_Co_0.48_ and Fe_0.98_O phases appeared in the X-ray diffraction (XRD) patterns, indicating that this reaction could alter the reaction thermodynamics by lowering the enthalpy of the desorption reaction. The improvement of LiAlH_4_ after the addition of CoFe_2_O_4_ as a catalyst is also supported by Wei et al. [43]. It is exciting to note that they created CoFe_2_O_4_ using a thermal decomposition method and discovered that the starts of hydrogen release as compared to pure LiAlH_4_ (176 °C and 279 °C for the first two stages) were lowered by 103.3 °C and 97.9 °C for the first two stages, respectively.

Many studies have observed that rare earth metals such as Nb, Y and La exhibited excellent hydriding and dehydriding kinetics of MgH_2_ [44,45]. Additionally, adding 10 wt.% LaFeO_3_ lowered the activation energies and started the hydrogen release of LiAlH_4_ as indicated by our earlier research [46]. A further study discovered that the desorption properties of LiAlH_4_ were greatly benefited by the freshly formed La or La-containing species, Fe and LiFeO_2_ that had been generated during the heating process. Based on the study by Zhou et al. [47], the reduction temperature in the endothermic peaks showed that the desorption properties of LiAlH_4_ were positively affected after the inclusion of LaF_3_. On the other hand, Xueping et al. [48] looked into how the addition of La_2_O_3_ affected the desorption properties of LiAlH_4_–NH_4_Cl. The result obtained shows that adding La_2_O_3_ to LiAlH_4_–NH_4_Cl will increase the rate of hydrogen release while reducing the starting time.

In this context, with the aim of combining La and Co, Cobalt lanthanum oxide (LaCoO_3_) was introduced to prepare LaCoO_3_–LiAlH_4_ via the ball-milling method to improve the hydrogen storage properties of LiAlH_4_. Interestingly, various weight percentages of LaCoO_3_ were investigated to study the catalytic effects and the microstructure of hydrogen storage properties of LiAlH_4_. To the best of the author’s knowledge, no research on LaCoO_3_ and LiAlH_4_ performance has been published.

## 2. Materials and Methods

In this study, LaCoO_3_ was used as an additive and was synthesized using the solid-state method as discussed in our previous research [49]. A total of 0.121 g of citric acid (≥98% pure; Sigma Aldrich, St. Louis, MO, USA), 0.081 g of lanthanum oxide (≥99.9% pure; Aldrich Chemical Compound, Milwaukee, WI, USA) and 0.040 g of pure cobalt oxide (99.99% pure, Sigma Aldrich) were ground in an agate mortar and calcined at 950 °C in a furnace for 5 h. Different weight percentages of pure LaCoO_3_ (5, 10, 15 and 20) were milled in a planetary ball mill (NQM–0.4) with LiAlH_4_ (95% pure, Sigma Aldrich) for an hour (15 min milling time, 2 min resting time with 3 cycles) at a rotational speed of 400 rpm with a ball to powder ratio of 40:1. To prevent the reaction of samples with oxygen and moisture, all operations were conducted in an argon atmosphere glove box (MBRAUN UNIlab, Germany) containing low concentrations of O_2_ (<0.1 ppm) and H_2_O (<0.1 ppm) and high-purity Ar (99.99%).

**Figure 1:**
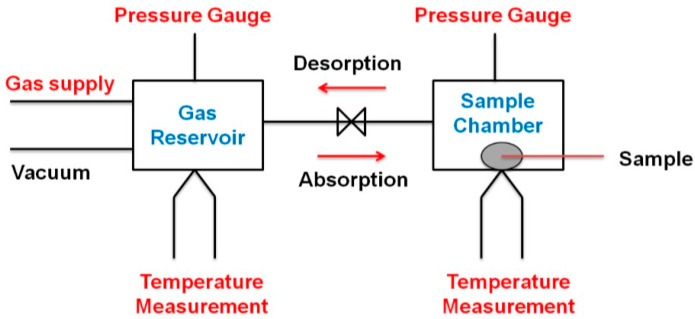
Schematic diagram showing the working principles of the Sieverts apparatus for determining hydrogen uptake/release.

The Gas Reaction Controller (GRC) evaluates quantitative analysis of the gas–solid reaction. It introduces a controlled amount of gas into the reaction chamber that holds a specimen. The pressure of the gas needs to be monitored while the temperature of the chamber is held constant or slowly changed. The instrument is connected to a computer and controlled by software (GrcLV), which performs fully automatic operations. Figure 1 below shows a schematic diagram of the working principles of the Sieverts apparatus. To determine the decomposition temperature of each sample, 150 mg of the materials were inserted into the sample holder and were heated at a rate of 5 °C/min from 30 to 250 °C. In the meantime, the desorption kinetics for all of the samples were heated for 80 min at 90 °C. This experiment was conducted by using Sievert-type apparatus (Advanced Materials Corporation, Pittsburgh, PA, USA).

Differential thermal analysis of the prepared samples was carried out using differential scanning calorimetry (Mettler Toledo, Columbus, OH, USA TGA/DSC 1) between 30–300 °C with a heating rate of 15, 20, 25 and 30 °C/min. Alumina crucibles were used as reference material. Samples weighing 6–8 mg were put into the crucible. The crucible was then sealed in a glass bottle to avoid the oxidation of the samples during the transfer from the glove box to the DSC apparatus. After the sample was placed inside the DSC, the gas inside the DSC was purged and constant argon flow (50 mL) was supplied. The designation of phase and composition of the samples were recorded by using XRD using Cu-Kα radiation at room temperature at a scanning range of 20° < 2θ < 80°. To prevent the oxidation of the samples, a small amount of the sample was spread uniformly on the sample holder and covered with scotch tape and followed by sealing with plastic wrap as described in our previous studies [50,51]. Fourier transform infrared (FTIR) spectroscopy spectra were collected at room temperature at a resolution of 4 cm^−1^ from 800 to 2000 cm^−1^. For FTIR, the samples were placed in microcentrifuge tubes (1.5 mL) during transportation from the glove box. The samples were placed on the FTIR, and the measurement was analyzed for a few seconds. We had additionally taken some precautionary steps so that the exposure of the samples to the air is minimized. Meanwhile, the surface configuration of the samples was analyzed using scanning electron microscopy (SEM; JEOL, Akishima, Tokyo, Japan (JSM-6360LA). Before each sample was analyzed, the samples were prepared on carbon tape and sprayed with a gold spray in a vacuum. Next, the samples were put in a sample container during the transfer process from the glove box to the SEM apparatus to prevent the samples from oxidizing. During the sample investigation, the samples were put inside the SEM under a vacuum state.

## 3. Results and Discussion

### 3.1. Onset Desorption Temperature

**Figure 2:**
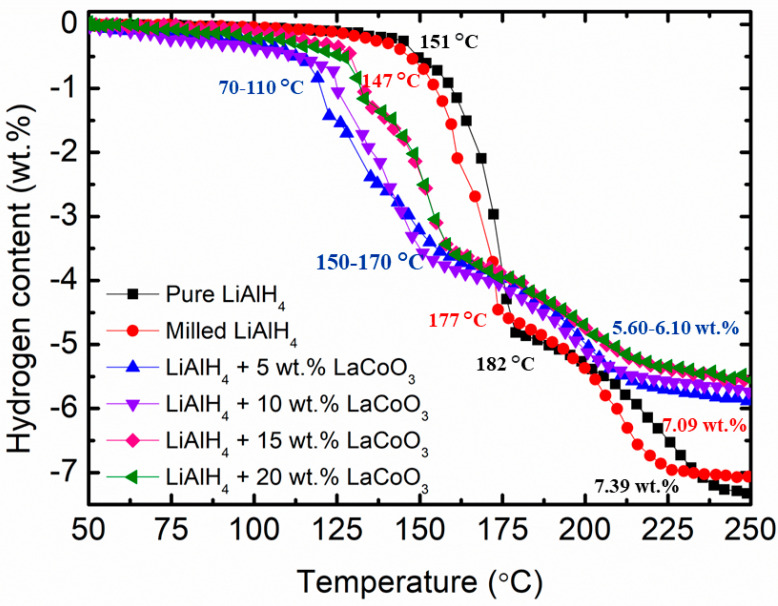
Onset desorption temperature of pure LiAlH_4_, milled LiAlH_4_ and LiAlH_4_ + x wt.% LaCoO_3_ (x = 5, 10, 15 and 20).

The hydrogen release curves with temperature are shown in Figure 2 for pure LiAlH_4_, milled LiAlH_4_ and LiAlH_4_ with different weight percents of LaCoO_3_. A remarkable low-temperature shift was observed for hydrogen released from LiAlH_4_ in the presence of LaCoO_3_. For pure LiAlH_4_, the first and second stages began at 151 °C and 182 °C, respectively with 7.39 wt.% of H_2_ released. However, after LiAlH_4_ was milled for an hour, the starting temperature release could be decreased from 151 °C to 147 °C and from 182 °C to 177 °C for the first and second stages, respectively, with 7.09 wt.% of the total H_2_ released. It is validated that the milling of the samples used has a positive effect on the peak of the desorption temperature of LiAlH_4_. This statement was also proven by Liu et al. [36]. Theoretically, LiAlH_4_ decomposes according to the following reactions [52,53]:

For the first stage,

3LiAlH_4_ → Li_3_AlH_6_ + 2Al + 3H_2_ (1)

and for the second stage,

Li_3_AlH_6_ → 3LiH + Al + 3/2H_2_ (2)

However, the temperature of onset desorption compared with milled LiAlH_4_ was reduced from 147 °C to 98 °C and from 177 °C to 153°C for the first and second stages, respectively, with 6.01 wt.% of H_2_ released when 5 wt.% of LaCoO_3_ was added. The desorption temperature drops to 70 °C and 105 °C for the first stage and 156 °C and 164 °C for the second stage, respectively, when 10 wt.% and 15 wt.% of LaCoO_3_ are added. The total H_2_ released for LiAlH_4_ + 10 wt.% LaCoO_3_ and LiAlH_4_ + 15 wt.% LaCoO_3_ were 5.86 wt.% and 5.72 wt.%, respectively. Increasing the amount of LaCoO_3_ to 20 wt.% also lowered the onset desorption temperature by 45 °C and 13 °C compared with pure LiAlH_4_ for the first and second stages, respectively. In spite of that, the amount of hydrogen released also decreased to 5.67 wt.%. This happens due to the zero hydrogen content in the LaCoO_3_; this outcome also has been testified by Xia et al. [54] and Ahmad et al. [55]. Table 1 summarizes the onset desorption temperature, hydrogen content and total hydrogen loss for pure LiAlH_4_, milled LiAlH_4_ and different weight percentages of LaCoO_3_ with LiAlH_4_ composites.

### 3.2. Isothermal Desorption Kinetics

**Figure 3:**
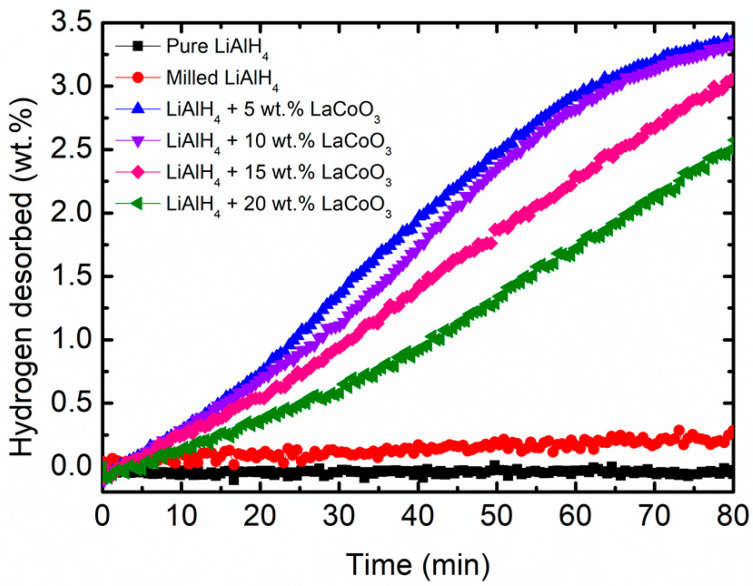
Isothermal desorption kinetics of pure LiAlH_4_, milled LiAlH_4_ and LiAlH_4_ + x wt.% LaCoO_3_ (x = 5, 10, 15 and 20).

For practical applications, the desorption rate of hydrogen storage materials is a crucial characteristic. Hence, Figure 3 below shows the isothermal desorption kinetics for LiAlH_4_ with different weight percents of LaCoO_3_ composites compared to pure LiAlH_4_ and milled LiAlH_4_ at 90 °C within 80 min. Under the same conditions, the LiAlH_4_ + 5 wt.% LaCoO_3_ systems desorbed 3.37 wt.% of H_2_. Increasing the amount of LaCoO_3_ to 10 wt.% also released 3.37 wt.% of H_2_ under the same period. However, slightly faster desorption kinetics can be observed for LiAlH_4_ + 5 wt.% LaCoO_3_ systems within 30 min at 90 °C. Meanwhile, LiAlH_4_ + 15 wt.% LaCoO_3_ and LiAlH_4_ + 20 wt.% LaCoO_3_ composites release approximately 3.06 wt.% and 2.50 wt.% of H_2_, respectively. The individual LiAlH_4_ releases less than 0.30 wt.% under the same conditions. This has proven that after the addition of LaCoO_3_, the isothermal desorption kinetics are 10 times faster than the unsubstituted LiAlH_4_. The next characterization was optimized to be 10 wt.% of LaCoO_3_. This is because the LiAlH_4_ + 10 wt.% LaCoO_3_ system decomposes at lower temperatures for the first stages compared to another amount of LaCoO_3_. Even though 5 wt.% also performed well for the isothermal desorption properties, the onset desorption temperature for 10 wt.% of LaCoO_3_ compared to that of LiAlH_4_ + 5 wt.% LaCoO_3_ (98 °C) decreased by 28 °C for the first stages, which indicates that the temperature for the LiAlH_4_ + 5 wt.% LaCoO_3_ is quite high for the desorption process to occur. Sulaiman and Ismail [56] also studied different weight percentages of SrFe_12_O_19_ (10, 20 and 50) on the thermal desorption of LiAlH_4_. A further study relating the catalytic effect of SrFe_12_O_19_ on the hydrogen storage performance of LiAlH_4_ was performed using 10 wt.% of SrFe_12_O_19_. Next, the effects of 5, 10, 15 and 20 wt.% of CoTiO_3_ were investigated on the onset desorption temperature of LiAlH_4_ [57]. However, 10 wt.% of CoTiO_3_ was chosen as an optimal amount to explore the desorption properties of LiAlH_4_. Thus, based on the discussion above, 10 wt.% of LaCoO_3_ was chosen for further characterization.

### 3.3. Activation Energy

**Figure 4:**
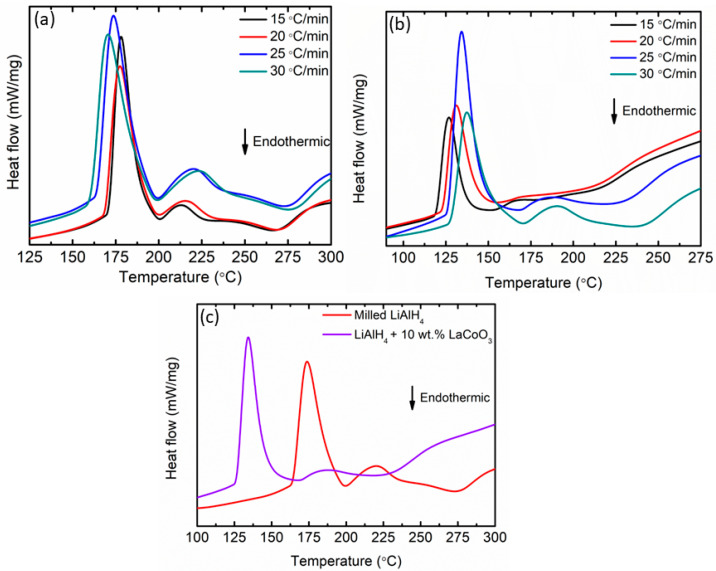
DSC traces of (**a**) milled LiAlH_4_, (**b**) LiAlH_4_ + 10 wt.% LaCoO_3_ at various heating rates and (**c**) DSC traces at 25 °C/min of milled LiAlH_4_ and LiAlH_4_ + 10 wt.% LaCoO_3_.

The DSC traces for milled LiAlH_4_ and LiAlH_4_ + 10 wt.% LaCoO_3_ are included in Figure 4a,b, respectively. The figure below indicates that as the heating rate increases, the temperature also increases. Figure 4c exhibits the comparison for milled LiAlH_4_ and LiAlH_4_ + 10 wt.% LaCoO_3_ at a heating rate of 25 °C/min. For milled LiAlH_4_, there are four peaks (two exothermic peaks and two endothermic peaks) [58,59]. At 174 °C, the first exothermic peak can be regarded as being caused by LiAlH_4_ interacting with the surface hydroxyl impurities. These characteristics are due to the presence of surface hydroxyl impurities in the alanate powder, as already described in a previous study [60]. The second exothermic peak at 220 °C was directly related to the decomposition of LiAlH_4_ to Li_3_AlH_6_ and Al (as in Equation (1)) and the second endothermic peak at 273 °C was believed to happen due to the decomposition of Li_3_AlH_6_ to LiH and Al (as in Equation (2)). In contrast with the unsubstituted samples, the temperature of each peak dropped after the inclusion of 10 wt.% LaCoO_3_. The first exothermic and endothermic peaks were 134 °C and 167 °C, respectively. Further heating led to additional exothermic and endothermic peaks at 188 °C and 242 °C, respectively. It has been shown that the addition of 10 wt.% LaCoO_3_ has shifted the peaks to a lower temperature for each reaction, proving that the desorption kinetics performance of LiAlH_4_ was improved. This could be associated with the fact that the introduction of 10 wt.% LaCoO_3_ leads to significant particle size refinement of LiAlH_4_. The lower desorption peaks of the composite can effectively contribute to the kinetic improvement of LiAlH_4_ via the addition of 10 wt.% LaCoO_3_. Furthermore, LaCoO_3_ is reported to have a hardness of 6.5 Mohs compared with the 4.0 Mohs of LiAlH_4_ [61]. The hardness of the LaCoO_3_ had thus broken the LiAlH_4_ particles. Therefore, smaller particle size could enhance the kinetic enhancement and lower the activation energy of the LiAlH_4_. The enhancement in the desorption kinetics performance is related to the energy barrier for hydrogen released from LiAlH_4_.

**Figure 5:**
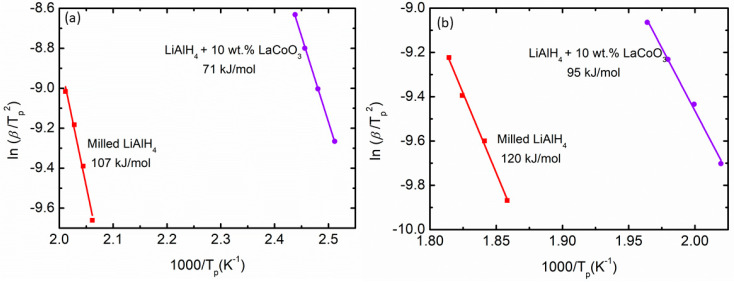
Activation energies of milled LiAlH_4_ and LiAlH_4_ + 10 wt.% LaCoO_3_ for (**a**) first stages and (**b**) second stages.

In order to obtain kinetic information of the hydrogen desorption from milled LiAlH_4_ and LiAlH_4_ + 10 wt.% LaCoO_3_, the activation energies (E_A_) were examined using the Kissinger equation, as shown in Equation (3) below:

ln [β/T_p_^2^] = −E_A_/RT_p_ + A (3)

where A = linear constant, R = gas constant, T_p_ = peak temperature in the DSC curve and β = heating rate. The activation value can be obtained by analyzing the slope of the Kissinger plot, ln (β/T_p_^2^) vs. 1000/T_p_. The first stage of E_A_ was applied to the decomposition of LiAlH_4_ as shown in Figure 5a while the second stage of E_A_ was applied to the decomposition of Li_3_AlH_6_ as illustrated in Figure 5b. From the figure, the E_A_ are 107 kJ/mol and 71 kJ/mol for milled LiAlH_4_ and LiAlH_4_ + 10 wt.% LaCoO_3_, respectively. For the second stage, milled LiAlH_4_ has an E_A_ of 120 kJ/mol and this value decreased by 25 kJ/mol after the addition of 10 wt.% LaCoO_3_ (95 kJ/mol). This proves that adding LaCoO_3_ as an additive could lower the E_A_ of LiAlH_4_ for both stages. A previous study by Li et al. [62] exposed that the E_A_ of LiAlH_4_ are 78.2 kJ/mol and 90.8 kJ/mol, 38 kJ/mol and 42.2 kJ/mol lower than pure LiAlH_4_ for the first and second stages, respectively, after the addition of K_2_TiF_6_. Furthermore, Zhang et al. [63] revealed that the E_A_ was reduced by 18 kJ/mol for the first stages and 73.3 kJ/mol compared to milled LiAlH_4_ (90.6 kJ/mol and 144.7 kJ/mol) after adding Li_2_TiO_3_. Adding SrFe_12_O_19_ also reduced the E_A_ from 103 kJ/mol to 76 kJ/mol for the first stage and from 111 kJ/mol to 96 kJ/mol for the second stage compared with milled LiAlH_4_ [56]. Based on this discussion, it can be concluded that adding LaCoO_3_ could lower the E_A_ of LiAlH_4_. Owing to this reduction of the E_A_, the desorption kinetics of LiAlH_4_ are enhanced.

### 3.4. Morphology and Microstructures

**Figure 6:**
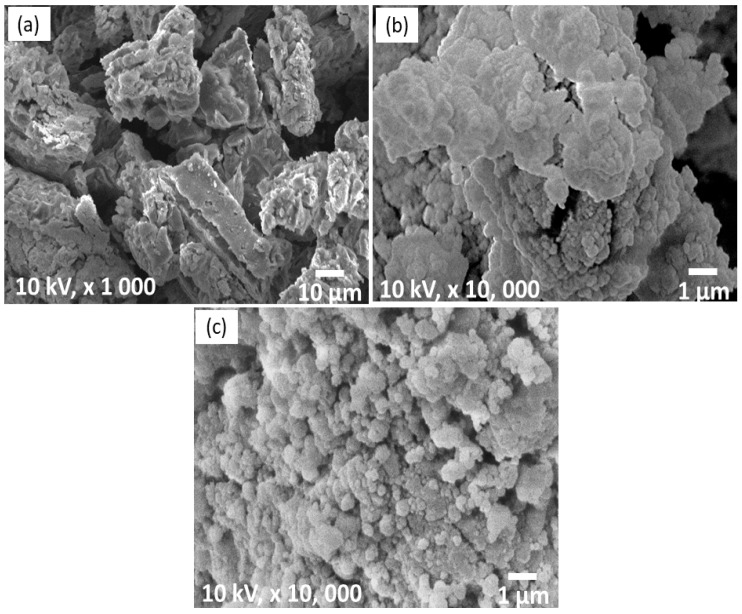
SEM images of (**a**) pure LiAlH_4_, (**b**) milled LiAlH_4_ and (**c**) LiAlH_4_ + 10 wt.% LaCoO_3_.

Figure 6 illustrates the SEM micrographs of pure LiAlH_4_, milled LiAlH_4_ and LiAlH_4_ + 10 wt.% LaCoO_3_. As shown in Figure 6a, the particles of pure LiAlH_4_ are shaped like blocks and are coarse, as reported by a previous study [64]. After LiAlH_4_ was milled for an hour, the particles became agglomerated and inhomogeneous, as shown in Figure 6b and proven by Ares and co-workers [65]. However, after the addition of LaCoO_3_, the particle size became small compared to pure LiAlH_4_ and milled LiAlH_4_ (Figure 6c). This result is consistent with earlier research showing that adding an additive or catalyst can reduce the particle size of LiAlH_4_ [66]. In addition, after LiAlH_4_ was doped with MnFe_2_O_4_, the samples decreased in particle size, resulting in more grain boundaries and increased surface area, which enhanced the desorption properties of LiAlH_4_ [67]. Therefore, it can be concluded that after the addition of 10 wt.% LaCoO_3_ through the ball-milling method, a smaller particle size can be observed, which may benefit the desorption behavior of the LiAlH_4_.

### 3.5. Particles Size Distribution

**Figure 7:**
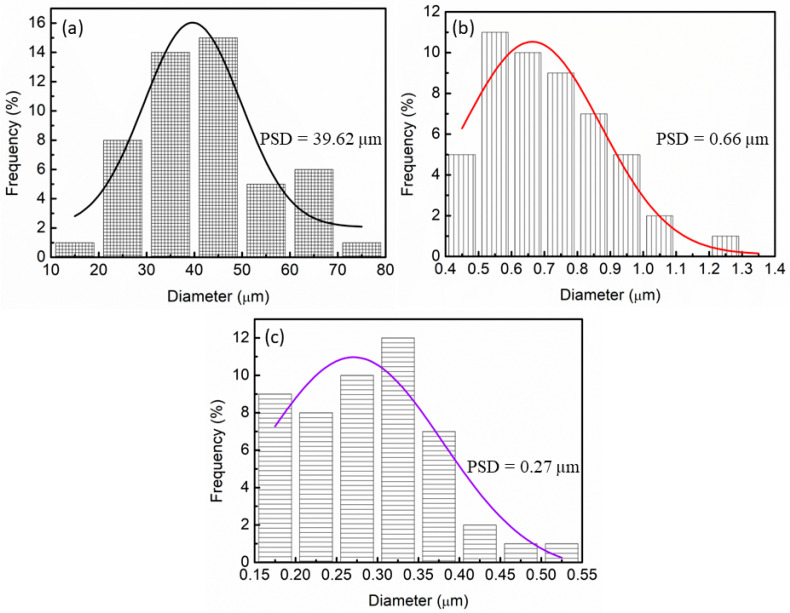
Particle size distributions of (**a**) pure LiAlH_4_, (**b**) milled LiAlH_4_ and (**c**) LiAlH_4_ + 10 wt.% LaCoO_3_.

The data plotted in Figure 7 show the average particle size distribution (PSD) of composites with and without the addition of LaCoO_3_. For pure LiAlH_4_ (as in Figure 7a), the particle size distribution calculated using ImageJ was 39.62 µm. After an hour of the milling process of LiAlH_4_, the samples improve the quality of the powders in terms of reduction in the particle size, as shown in Figure 7b. The particle size of milled LiAlH_4_ was reduced to 0.66 µm. However, the particle size of LiAlH_4_ + 10 wt.% LaCoO_3_ is smaller if compared to the unsubstituted samples previously described in the SEM part. The average particle size of LiAlH_4_ + 10 wt.% LaCoO_3_ was 0.27 µm, as shown in Figure 7c. In summary, the progressive phenomenon of reduction in particle size is reached, indicating that the inclusion of LaCoO_3_ using the ball-milling method improved the desorption performance of LiAlH_4_.

### 3.6. Phase Structure

**Figure 8:**
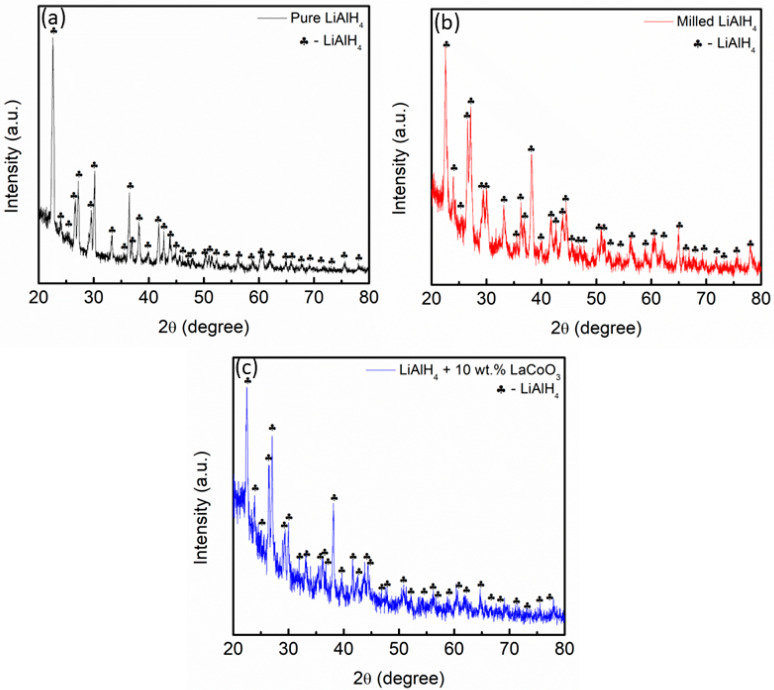
XRD pattern of (**a**) pure LiAlH_4_, (**b**) milled LiAlH_4_ and (**c**) LiAlH_4_ + 10 wt.% LaCoO_3_.

The XRD patterns of pure LiAlH_4_ and LiAlH_4_ after being milled for an hour are displayed in Figure 8a,b, respectively. At this stage, all the peaks correspond to LiAlH_4_, which perfectly matches the standard pattern of LiAlH_4_ (JCPDS card no. 73-461). However, the intensity of the sample lowered compared to pure LiAlH_4_. According to these patterns, the milling process will result in a reduction in the intensity of the peaks. A previous study by Rahmaninasab and colleagues [68] revealed that the milling time causes the intensity to be reduced. This is due to the increase in the lattice strain and internal energy during the milling process, as revealed by Dittrich et al. [69]. This also demonstrates that LiAlH_4_ is stable during the milling process, as stated in the previous studies [32,70,71]. The XRD pattern of LiAlH_4_ + 10 wt.% LaCoO_3_ is presented in Figure 8c. It was seen that the peaks related to LiAlH_4_ become broad and had a lower intensity when compared to unsubstituted LiAlH_4_. No new peaks or peaks for LaCoO_3_ were detected during the milling process of LaCoO_3_.

**Figure 9:**
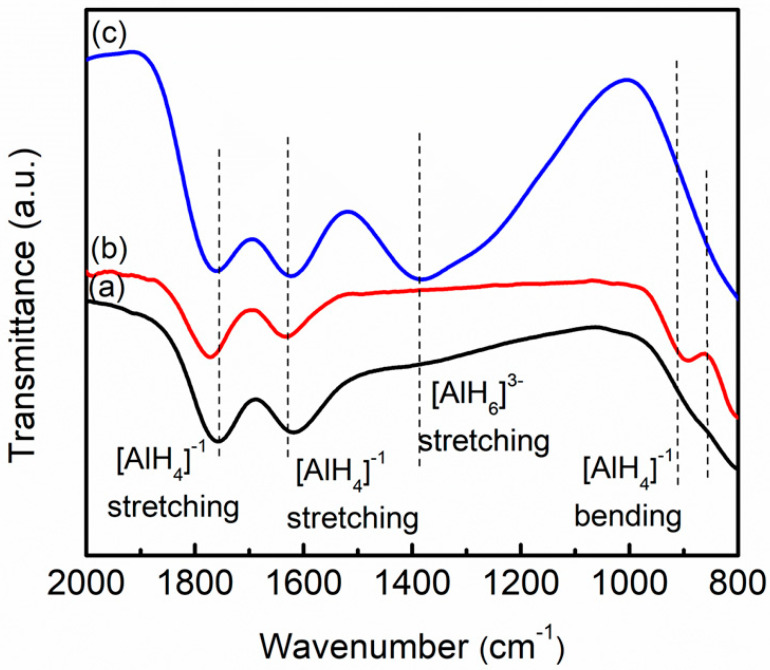
FTIR pattern of (a) pure LiAlH_4_, (b) milled LiAlH_4_ and (c) LiAlH_4_ + 10 wt.% LaCoO_3_.

The FTIR spectra were carried out to confirm the presence of 10 wt.% LaCoO_3_, as indicated in Figure 9. Two regions of active IR peaks that are the bending mode and the stretching mode were detected in the range of 800–900 cm^−1^ and 1600–1800 cm^−1^ [72], respectively, for pure LiAlH_4_, milled LiAlH_4_ and LiAlH_4_ + 10 wt.% LaCoO_3_. After the addition of 10 wt.% LaCoO_3_, a new peak was detected at 1383 cm^−1^, suggesting that LiAlH_4_ was decomposed to Li_3_AlH_6_ and Al during the ball-milling process. A previous study led by Shen et al. [73] also revealed that the peaks that appear around 1400 cm^−1^ represent the stretching peak of Li_3_AlH_6_. Furthermore, after the addition of NiFe_2_O_4_, Wei et al. [43] discovered a weak stretching mode at about 1404 cm^−1^ which belongs to the Al–H stretching of [AlH_6_]^3−^.

**Figure 10:**
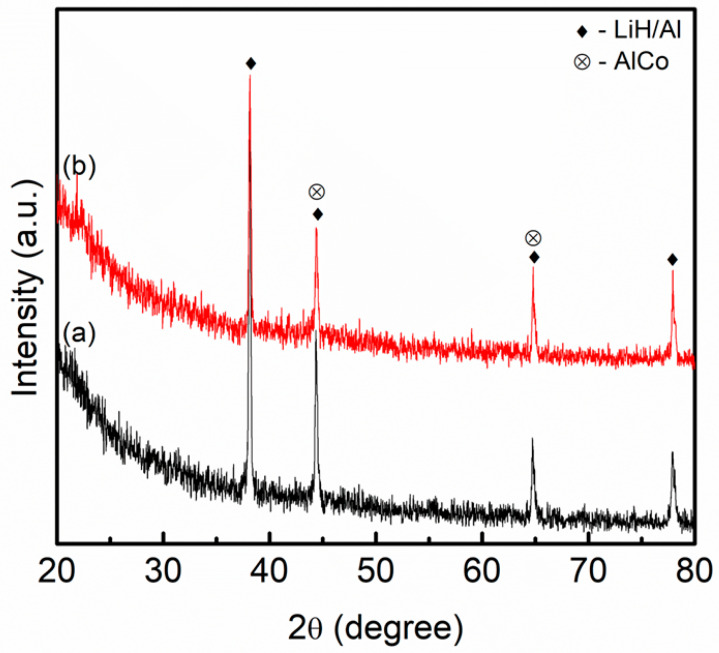
XRD pattern of (a) LiAlH_4_ + 10 wt.% LaCoO_3_ and (b) LiAlH_4_ + 30 wt.% LaCoO_3_ after desorption at 250 °C.

To observe the change of phase structure of the LiAlH_4_ with LaCoO_3_ during the desorption process, an XRD scan has been conducted from 20° to 80°. As shown in Figure 10a below, after LiAlH_4_ + 10 wt.% LaCoO_3_ was heated, LiH and Al peaks were found, indicating that Equations (1) and (2) had occurred. A new peak for AlCo was identified, proving that the reaction of LiAlH_4_ and LaCoO_3_ may have occurred during the heating process. No peaks for La or La-containing species were detected as displayed in Figure 10a. However, after the amount of LaCoO_3_ was increased to 30 wt.% (as shown in Figure 10b), the same peaks for AlCo and LiH/Al were spotted.

From the above discussions, the enhancement of LiAlH_4_ may be ascribed to a few factors. The new active species of AlCo formed after the heating process acts as a real additive in enhancing the hydrogen storage performance of LiAlH_4_. Additionally, it has been evidenced that Co additives may benefit the desorption properties of light metal complex hydrides [74,75]. In addition, Lv and colleagues [76] managed to show that the Co element significantly improves the hydrogen storage performance of the alloy. Other than that, Ali et al. [77] indicated that the formation of Al–Co alloys during the heating process of NaAlH_4_ and CoTiO_3_ is the cause of the enhanced desorption behavior of NaAlH_4_. Furthermore, the starting hydrogen released by NaAlH_4_ compared to milled NaAlH_4_ (200 °C) decreased by ~65 °C after the addition of CoTiO_3_. Moreover, hydrogen storage desorption of LiAlH_4_ was improved by the presence of CoFe_2_O_4_ nanoparticles [42]. The start of hydrogen release dropped to 65 °C at the first stage and the activation energy lowered by 42.4 kJ/mol in the presence of CoFe_2_O_4_ nanoparticles. One of the reasons for the enhancement of LiAlH_4_ is due to the formation of Al–Co phases during the heating process of LiAlH_4_ and CoFe_2_O_4_ composites. However, no peak for La or La-containing species (such as La–O, Co-La or Al-La) was detected due to the low amount of LaCoO_3_ in the composite or La being amorphous. This result was consistent with our previous study [46]. Hence, it is possible to speculate in situ formation of AlCo and La or La-containing species during the heating process attributed to improving the desorption kinetics of LiAlH_4_.

## 4. Conclusions

In this paper, thermal desorption of LiAlH_4_ has been studied with and without the addition of different weight percentages of LaCoO_3_ additive (5, 10, 15 and 20). It was found that the addition of 10 wt.% LaCoO_3_ is an effective additive which substantially lowered the onset desorption temperature by 81 °C and 26 °C for LiAlH_4_ compared with pure LiAlH_4_ for the first two stages, respectively. Furthermore, the desorption kinetics are 10 times faster than the unsubstituted LiAlH_4_ at 90 °C for 80 min. The LaCoO_3_ additive considerably reduced the activation energies from 107 kJ/mol to 71 kJ/mol for the first stages and from 120 kJ/mol to 95 kJ/mol for the second stages. From our observation, the morphology of LiAlH_4_ + 10 wt.% LaCoO_3_ shows that the particle size becomes smaller and less agglomerate compared to the unsubstituted LiAlH_4_. The result of particle size distribution also proved that the addition of LaCoO_3_ additive reduced the particle size to 0.27 µm, 39.35 µm lower than pure LiAlH_4_ and 0.39 µm reduced from milled LiAlH_4_. Furthermore, the formation of new phases (AlCo and La or La-containing species) have a notable impact on enhancing the hydrogen storage behavior of LiAlH_4_, which can be determined by observing the reaction between LiAlH_4_ and LaCoO_3_ during the heating process.

## Figures and Tables

**Table 1:** Onset desorption temperature, hydrogen content and hydrogen loss from each sample.

Samples	Onset Desorption Temperature (1st Stage) (°C)	Onset Desorption Temperature (2nd Stage) (°C)	Hydrogen Content (wt.%)	Hydrogen Loss (wt.%)
Pure LiAlH_4_	151	182	7.39	-
Milled LiAlH_4_	147	177	7.09	0.30
LiAlH_4_ + 5 wt.% LaCoO_3_	98	153	6.01	1.38
LiAlH_4_ + 10 wt.% LaCoO_3_	70	156	5.86	1.53
LiAlH_4_ + 15 wt.% LaCoO_3_	105	164	5.72	1.67
LiAlH_4_ + 20 wt.% LaCoO_3_	106	169	5.67	1.72

## Data Availability

The data presented in this study are available on request from the corresponding author.
